# Social dynamics of short term variability in key measures of household and community wellbeing in Bangladesh

**DOI:** 10.1038/s41597-019-0128-0

**Published:** 2019-07-17

**Authors:** Md. Ehsanul Haque Tamal, Andrew R. Bell, Mary E. Killilea, Patrick S. Ward

**Affiliations:** 1 International Food Policy Research Institute, Dhaka, Bangladesh; 2 New York University, New York, USA; 3 Duke Kunshan University, Kunshan, China

**Keywords:** Environmental economics, Agriculture

## Abstract

High-frequency social data collection may facilitate improved recall, more inclusive reporting, and improved capture of intra-period variability. Although there are examples of small studies collecting particular variables at high frequency in the social science literature, to date there have been no significant efforts to collect a wide range of variables with high frequency. We have implemented the first such effort with a smartphone-based data collection approach, systematically varying the frequency of survey task and recall period, allowing the analysis of the relative merit of high-frequency data collection for different key variables in household surveys. This study of 480 farmers from northwestern Bangladesh over approximately one year of continuous data on key measures of household and community wellbeing could be particularly useful for the design and evaluation of development interventions and policies. While the data discussed here provide a snapshot of what is possible, we also highlight their strength for providing opportunities for interdisciplinary research in the household agricultural production, practices, seasonal hunger, etc., in a low-income agrarian society.

## Abstract (summary)

**Table:** 

Design Type(s)	observation design • time series design • behavioral data analysis objective
Measurement Type(s)	Quality of Life
Technology Type(s)	crowd-sourced data generation
Factor Type(s)	frequency • geographic location
Sample Characteristic(s)	Homo sapiens • Rangpur District • rural area

Machine-accessible metadata file describing the reported data (ISA-Tab format)

## Background & Summary

Conventional household surveys typically use multiple visits with a large time gap between visits to construct longitudinal data^1^. These data typically suffer from recall bias and lost intra-period variation, as respondents are asked in one sitting to recall events or outcomes that have transpired during the entire period between survey interviews^2,3^. Furthermore, these surveys are very expensive to conduct^4^, with large enumeration teams visiting respondents, thus increasing the propensity for enumeration or data-entry error. The advent of Computer-assisted Personal Interviewing (CAPI) via laptop, tablet or smartphone, has made data collection cheaper, more reliable, and ultimately more efficient^5^. But even with CAPI tools at their disposal, these large household surveys require enumerators to engage with participants for an extended period of time to complete lengthy questionnaires, which can result in respondent fatigue, and consequently, poor quality and unreliable data^3^. It is also frequently difficult to collect data from remote areas and to conduct surveys during natural disasters, political disorder, etc., all of which are frequent in developing country contexts. The dataset described in this note provides some of the first evidence on the feasibility of an alternative to these costly, infrequent, difficult-to-conduct surveys. In particular, the data reported here were collected over the course of 50 weeks at relatively high frequency using smartphones. To ameliorate concerns of respondent fatigue, the long-form survey instrument (based on an integrated household survey instrument) was decomposed into small ‘microtasks’ that participants could address in the course of 5–10 minutes each. The study encouraged continued engagement and active participation in the data collection efforts through ‘micropayments’ that were awarded upon the successful submission of the microtasks, as well as credits toward the ownership of the smartphone. By putting the smartphones directly into the respondents’ hands, our study could also capture data easily from those at the focus of study, even those in remote places where access is limited or unreliable.

This dataset contains approximately one year’s worth of high frequency data containing information on a wide variety of experiences, inputs, and outcomes such as agricultural production and practices; experiences with income shocks and climatic events; household income, expenditure, consumption, labor, employment; migration; housing and sanitation; information and technology; as well as basic demographic and household characteristics.

The data collection was conducted with an Android based smartphone using a customized launcher for Open Data Kit (ODK) – an open-source, user-friendly, and easily deployable set of tools supporting data collection, visualization, and sharing, without the complications of setting up and maintaining one’s own servers^6^. Survey were translated into Bangla and pretested on the application platform. The implementing partner, WIN Incorporated, assisted in the selection and training of participants, distribution of the smartphones, and provided oversight over the data collection process. A mobile operator, Banglalink, provided SIM cards, talk time, and mobile data to support the farmers as rewards based on their responses on the questions.

The data collection covered 480 farm households drawn from 40 villages in two subdistricts of Rangpur district in northwestern Bangladesh, selected using a multistage sampling technique. Farmers were selected randomly from a sampling frame that was prepared in consultation with the local agricultural extension officers, specifically focused on farmers’ literacy and the likelihood that they would emerge as an early adopter of smartphone technologies. Participants responded regularly to 46 different survey tasks along a one-year period, with the frequency with which they received each task randomized to be either weekly, monthly, or seasonally (see Methods).

The design of this study allows measurement of recall bias and missed intra-period variation. Additionally, it allows identification of the most appropriate frequencies (weekly, monthly, or seasonally) to assess consumption, spending, labor, or other aspects of life experience^7^. Further, the dataset allows examination of quantities commonly collected as point estimates – such as subjective well-being – as time series and distributions. Particular examples of possible uses of the dataset include analysis of (i) intra-annual food security dynamics, with data on grain storage and 24-hour recall food diaries, (ii) consumption responses to exogenous shocks such as climate events or changes in the labor market, or (iii) linkages from access and use of clean water to illness and missed work or school. In sum, we expect the dataset to provide a high-frequency, intra-annually resolved window into the dynamics of topics commonly summarized as single numbers in an integrated household survey, albeit for a small and purposively selected sample.

## Methods

### Participant selection

**Fig. 1:**
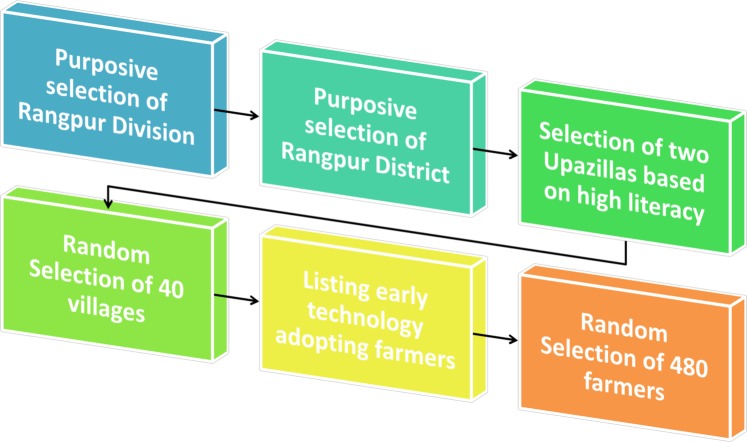
The multistage sampling scheme for participant selection.

Our sample was designed with a multistage sampling technique (Fig. 1) aimed at reaching farming households who were potential early adopters of smartphones. We selected Rangpur district in northwestern Bangladesh and based on the literacy rate reported in the Bangladesh National Census 2011, selected the two most literate upazillas (sub-districts) from the eight upazillas of Rangpur district, namely Mithapukur and Rangpur Sadar^8^. We then randomly selected 20 villages from each of these two upazillas, for a combined pool of 40 villages. Through our local implementing partner WIN Incorporated we assigned four local Points of Contact (POC) in each upazilla, who consulted with local agricultural extension officers to make a list (sampling frame) of technology early adopting farmers, who the officers deemed likely to be among the first to engage with smartphones. These farmer lists varied in length from 15 to 40 in different villages. We then randomly selected 8 to 16 farmers from these lists (proportionally depending on the length of the list) from different villages, for a total sample of 480 farmers. Over a series of half-day workshops in their home villages, these farmers were trained intensively to use the ODK application along with some other basic features of smartphone usage (see Survey Implementation).

### Data collection platform

The data collection process was designed on ODK Collect for use on Google’s open source Android operating system. It supports a form, survey, or algorithm into a sequence of input prompts that provide navigation logic, entry constraints, and repeating substructures^6^. The device distributed to the participants was the Symphony Roar V25 smartphone using Android 4.4.2, each of which were preloaded with the ODK application and a custom launcher “Data Exchange” developed by Nafundi (the developers of ODK; https://nafundi.com/). Participant responses were stored on ODK Aggregate server, a ready-to deploy server that hosts forms and submitted results. It aggregates collected data and provides standard interfaces to extract data such as spreadsheets. ODK Aggregate is implemented on Google’s App Engine and allows users to avoid the hassle of setting up one’s own reliable service^6^. Across our project we used 20 Aggregate servers to capture data from 20 unique phone setups (see Smartphone Setups).

### Smartphone setup

We structured our questionnaire as 46 short (5–10 minute) tasks, most of which were based on existing modules in the Bangladesh Integrated Household Survey^9^. Most of the survey items in these tasks are closed-ended with single or multiple selection options, while in a few cases participants were asked to input their answers in words or in numbers. Some questions asked them to take photos of places such as tubewells or latrines, or record a GPS location. While some tasks (for which relatively little intra-annual variation would be expected, such as information on farm plots or household members) were included only once in the experiment, most tasks were given to participants multiple times along the 50-week experiment at weekly, monthly, or seasonal frequencies. Additionally, many tasks included a ‘crowdsourcing’ component, in which the respondent would complete the task first for him- or herself, and then repeat the task for any friend, neighbor, or passerby; these participants were ideally selected on an approximately random basis, and their anonymity was ensured through the absence of personally identifying questions. The purpose of this crowdsourcing component in the study was to examine whether a crowdsourced sample might demonstrate a reduced selection bias, and thus better approximate a true representative sample, than our purposive sample of potential early adopters. Each crowdsourced task replicates the task completed by the sample respondent, with the additional request for basic demographic information (i.e., age, gender, literacy, education) at the beginning of the task. We do not assume that the same individuals are tracked over time in the crowdsourced sample. While this limits some uses of the crowdsourced sample, as it does not provide within-subject time series, it also means that the dataset includes a high-frequency cross-sectional sample that is not subject to panel conditioning (where repeated engagement with the same task affects the way a respondent performs it^10^). This inclusion possibly enables an identification of panel conditioning effects in our main sample, by providing an analogously high-frequency set of responses that are not completed repeatedly by the same respondents against which the main sample might be compared. The versions of each task included in the study are summarized in Online-only Table 1.

We constructed 20 unique smartphone setups (each given to 24 respondents, for a total of 480 participants in the sample). Each setup included exactly one version of each of the 46 tasks (e.g., Task 12, repeated monthly, not crowdsourced). We created these setups by first randomly assigning one version of each task (weekly, seasonally or monthly; crowdsourced or not) to each phone setup. Since versions with higher frequency (and those that included a crowdsource component) present both a higher respondent burden and a higher earning potential, and having the goal of standardizing both effort and earnings across setups, we then used a script (coded in Matlab) to make pairwise switches of task versions between smartphone setups until the Gini coefficient of earnings potential (from micropayments for participation in the survey throughout the duration of the project) across the phones fell below 0.001, indicating near perfect equality across setups^11^.

### Ethical approval

This study was reviewed and approved as a minimal risk application by International Food Policy Research Institute’s (IFPRI’s) Institutional Review Board (IRB #00007490, FWA #00005121). The IRB application number is 2015-49-EPTD-M, approved on 09/21/2015 under the title “Crowdsourcing Rural Data Collection via Android”. All research staff working directly with this research were required to have completed IFPRI’s CITI training course. Also, all field data collection staff were briefed in research ethics, including informed consent and data privacy. A written consent form (approved by the IRB) was signed by all the participants with one copy sent to IFPRI and one kept by the participants.

### Survey implementation

**Fig. 2:**
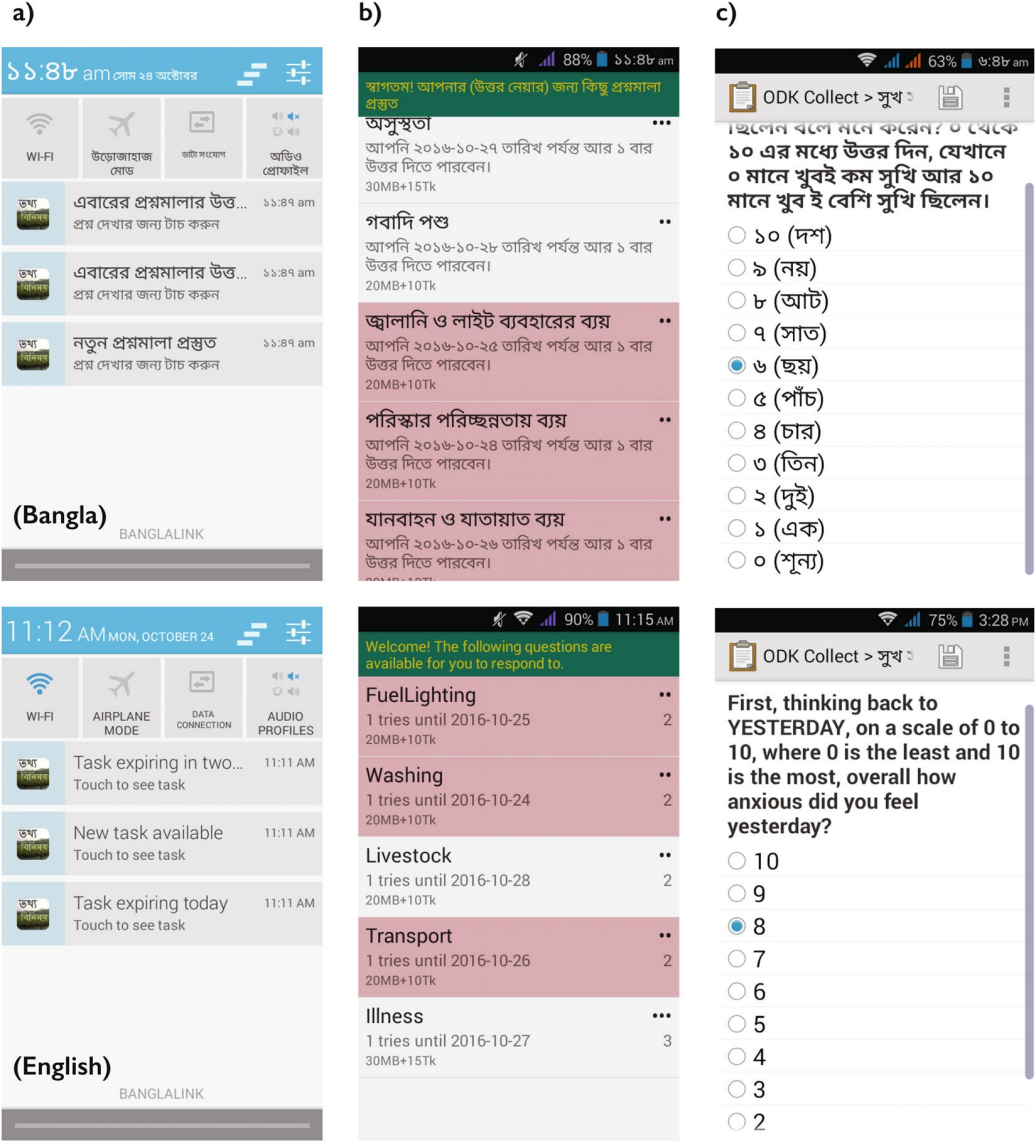
Survey task experience of participants. (**a**) Participants are notified via a push notification that tasks are available. (**b**) When participants tap the notification or the app, the Data Exchange interface appears. (**c**) From which Android ODK is launched for the individual tasks – adapted from Bell *et al*.^11^.

Data collection was facilitated by WIN Incorporated, via eight Points of Contact (POC) from different villages of Rangpur. We conducted a Training of Trainers (ToT) with the POCs to provide a full orientation with the ODK system. The ToTs are a way to prepare new trainers with appropriate background, skills and hands on experience on the survey system to provide farmers proper knowledge and technical assistance. Following these trainings, the POCs conducted training with small groups of farmers (consisting of the 8–16 farmer participants from a particular village) to orient them on the full data collection approach and timeline. We used a custom ODK launcher application called “Data Exchange” in order to streamline participation for the participants (Fig. 2), tested along with several survey questions in a pretest with a separate sample of farmers to check the user friendliness of the platform, comprehensiveness of the questions and participant comprehension. Following the outcomes from the pretesting, we developed a training guidebook (in Bangla with visual instructions) adjusted survey item wording per the pretesting findings.

**Fig. 3:**
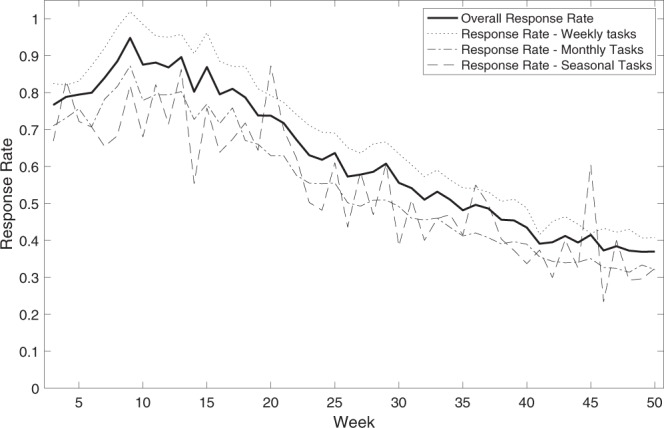
Response rates to weekly, monthly, and seasonal tasks along the length of the experiment.

WIN Incorporated had multiple layers of team management in place to maintain the quality of the work. The eight POCs reported to and were guided by two local field managers based in Rangpur, who in turn reported to senior staff at WIN Incorporated and the project leads. The full process was monitored and scrutinized by IFPRI with frequent random checks. POCs were in close contact with the participants during the early stage of the study to troubleshoot any issues they faced. A common early issue was the loss of installed apps and tasks due to a manual resetting of the device. Consequently, POCs were trained to reinstall all project software to address this. Engagement with the experiment varied along the course of data collection, with average response rates to tasks peaking at over 90% around the 10^th^ week of the experiment and declining to around 40% at the 50^th^ week of the experiment, with average response rates slightly higher for weekly tasks (Fig. 3).

## Data Records

Project data are stored in the Harvard data repository^12^ as standalone databases. The data can be categorized broadly into two types.

1. Non-repeated modules: For the most part, these are the demographic characteristics of the households, collected only once from the participants. There are three non-repeated modules: (a) Basic Information, (b) Household composition, and (c) Plots; b and c each contain nested data files that provide records for individual household members and plot level data, respectively.
2. Repeated Modules: These modules contain data on crops, production, consumption etc. A total of 97 files are available under this category including the nested data files. Typically, one repeated module has several nested data files, where nested files provide individual records for data types identified in the task (crops, animals, members, events, etc.); for example, the module “DrinkingWater” contains nested file named “DrinkingWater_source_level_2” to capture information about each of the different sources. The more complicated example “FertilizersAndPesticides” contains nested files:

- “FertilizersAndPesticides_plot_repeat_begin_level_2”,
- “FertilizersAndPesticides_crop_level_3”
- “FertilizersAndPesticides_group_crop_i_croptype_level_4”,
- “FertilizersAndPesticides_rep_fert_level_5”.

that include records for the plots treated, the kinds of crops treated in each plot, the specific crops within each crop type treated, and the specific treatments applied to these specific crops, respectively. The nested files are named with an underscore sign (“_”) and “level_#” after the main file names. A full list is presented in Online-only Table 2.

## Technical Validation

We have maintained data quality across the following steps: (1) Questionnaire translation into Bangla adopting standard survey questionnaire, (2) questionnaire and ODK platform pretesting, (3) Recruiting tech-savvy POCs and farmers likely to be early adopters of smartphone technology, (4) ToT and thorough training on the ODK platform, 5) Data checking and cleaning from STATA and R.

The majority of the survey questions were adopted from the Bangladesh Integrated Household Survey (BIHS)^9^ and modified as required to fit the smartphone modality. BIHS survey questions and their translations into Bangla had previously been tested, and the use of exactly the same translation increases the acceptability of our survey questions. We further pre-tested the comprehensiveness of the questions and farmer comprehension after training in a session conducted with approximately 10 farmers in the village of Manikgong, close to Dhaka. We used four different modules whose items spanned the full range of input modes in the experiment, including single selection, multiple selection, taking photos, capturing GPS, etc. This pretesting enabled us to test both the questionnaires and the ODK platform from the users’ perspective. As our sampled participants were chosen from among likely early adopters of smartphone technology, we observed that it was generally easy for them to familiarize themselves with the full process of answering questions and the overall functionality of the handsets. Moreover, the POCs were chosen from among university students with good understanding of technology, facilitating troubleshooting with the participants after the culmination of the training. We conducted a ToT with the POCs and total of 40 half day training sessions (one in each village) with the participants. Each training was conducted by two POCs and coordinated by a field manager along with a senior staff from WIN Incorporated. We prepared a training manual translated into Bangla which the POCs used in training the farmers, and which was distributed to the farmers for their own reference. The trainings covered a brief overview of our data collection project, basic mobile care issues, basic and advanced functions of the smartphone, orientation with the data collection application (“Data Exchange”), an introduction to various sample survey forms, an outline of different survey tasks, and self-practice with several survey forms. Once the participants started submitting data, we checked their submissions on a daily basis from the ODK aggregate servers and sent them micropayments (mobile data and talk-time facilitated by Banglalink). Following completion of the near-one-year pilot study, data were collated and minimally cleaned prior to archival. We did not alter any data (e.g., censoring or other treatment of outliers) and did not discard any cases, as the use of logical checks and input bounding available within the ODK platform prevented the occurrence (to our knowledge) of unusable data. Our experiment did not include a ground-truthing component, and so we are not able to directly compare the quality of responses made via smartphone to those that might have been made using a traditional survey approach.

## Usage Notes

### Participants’ identification access

Publicly available data from this project redacts identifiers such as International Mobile Equipment Identity (IMEI) numbers, device numbers, phone numbers, etc., that could be used to identify participants, either directly or as the result of deduction. All GPS coordinates that are included in the dataset have been modified with minor random noise to prevent participant identification, though participants’ modified GPS locations will provide a proportionate spatial measure that allows the user to analyze geographical relationships. No other personally-identifying data were collected, and hence the confidentiality and anonymity of the participants were fully ensured. Participants’ name and location-related information was collected as part of their informed consent, but those data were not published anywhere and were kept locked in a secure location at IFPRI’s Dhaka office with access only available to project researchers. We presume none of this will discourage any researcher to reuse the data. Any researcher requiring actual GPS coordinates, or any other personally-identifying information, is asked to contact the authors; sharing of any such data would require at a minimum ethical clearance from his/her institution, as well as signed data-use agreements that ensure the maintenance of respondent confidentiality and data security.

### Potential for double counting

Modules tasking participants to collect information on tubewells and latrines may include records in separate responses identifying the same locations, as many respondents may share the same irrigation facilities and reside in the same villages.

## Supplementary Information

### ISA-Tab metadata file

Download metadata file

### Supplementary Information

Supplementary File 1

Supplementary File 2

## Data Availability

The data are published as raw, without modification or recoding, as downloaded from the Google App Engine Servers, with the exception of GPS coordinates (see Usage Notes). Data from all unique phone setups and different frequencies were concatenated in single tables, and in some cases variables have been relabeled for clarity and consistency. The Matlab routines used to develop the initial smartphone setups and the summary information of the phone setups are included as Supplementary Files 1 and 2 to this publication.

## Online-only Tables

**Online-only Table 1:** Frequency and crowdsourcing of data collection.

Module	Description	Non-repeated	Seasonal	Monthly	Weekly	Crowdsourced
Basic Information	Age, religion, location, etc. of respondent	X				
Household Composition	Basic information on household members	X				
Employment	Household members’ employment, hours worked, wages, etc.		X	X	X	X
Information Tools	Use of information and communication technologies		X	X		X
Transportation Tools	Use of manual and mechanical sources of transportation		X	X		X
Work Animals	Ownership and utilization of draft animals		X	X		
Savings	Formal and informal savings		X	X		
Loans	Formal and informal loans		X	X		
Plots	Basic information on plots/ponds used for cultivation	X				
Crops	Listing of crops & estimating associated area, cultivation, cost, etc.		X			
Irrigation	Irrigation sources, method, cost of each crops		X	X	X	
Fertilizers and Pesticides	Use of chemicals, fertilizers, or pesticides		X	X	X	
Tools	Rental of any tools, machinery or draft animals for agricultural work		X			
Farm Labor	Labor done by household member or pay others to do any work on farm plots		X	X	X	
Agricultural Production	Agricultural inputs and harvests.		X	X	X	
Food Storage Capacity	Food storage for rice and wheat			X	X	
Agricultural Extension	Agricultural extension services such as advice on fertilizer, seed etc. received by households		X	X		
Agricultural Subsidies	Receipt of agricultural subsidy cards		X			
Livestock	Usage of livestock and poultry animals		X	X	X	
Fish Inputs	Inputs used for ponds		X	X		
Fish production	Production, consumption, revenues of fish on ponds & rivers		X	X	X	
Marketing of crop/animal production	Marketing of Agriculture, Livestock, and Fisheries		X	X	X	
Non-agricultural enterprise	Non-farm economic activities owned or operated by household members	X				
Food consumption	Food consumption of household members or friend, neighbor or other person		X	X	X	X
Non-food expenditure - Washing	Expenditures on washing and cleaning of household members		X	X	X	
Non-food expenditure - Transport	Expenditures on transport and travel by household members		X	X	X	
Non-food expenditure - Lighting	Expenditures on fuel and lighting by household members		X	X	X	
Non-food expenditure - Cosmetics	Expenditures on cosmetics and beauty products by household members		X	X	X	
Housing and Sanitation	Housing and Sanitation arrangements types, utilities, rents etc.	X				
Facilities	Transportation, hospital, bank, market etc. facilities around home	X				
Bad Shocks	Negative events or shocks experienced by household members, friend, neighbor, or other person		X	X	X	X
Good Shocks	Positive events or shocks experienced by household members, friend, neighbor, or other person		X	X	X	X
Safety Programs	Benefits received from social safety net programs		X			
Current Migrants	Migration of household members		X			
Remittance In	Remittances received by household		X	X		
Remittance Out	Remittances sent out to migrant family members		X	X		
Other income	Other sources of income beyond farm or other business		X	X		
Illness	Illness or injury of the household members, friend, neighbor, or other person		X	X	X	X
School attendance	School attendance of the household members, friend, neighbor, or other person		X	X	X	X
Subjective Well-being	Wellbeing (feeling, happiness, anxiety) of a family member, friend, neighbor, or other person		X	X	X	X
Tubewell Walk	Map out tubewells of the area including GPS location, pictures etc.	X				
Latrine Use	Access and utilization of latrine, type of toilet facility etc.	X				
Latrine Walk	Map out some latrines in the area including GPS location, pictures, facilities etc.	X				
Internet Use	Usage of smartphone and internet access			X	X	
Drinking water Diary	Use of drinking water by household members, friend, neighbor, or other person		X	X	X	X

**Online-only Table 2:** List of data files.

Module Name	Nested Files	
	Order	Name
Agricultural Extension Services	—	Form is not nested
Agricultural Production	1	Agricultural Production_level_1
	2	Agricultural Production_plot_repeat_begin_level_2
	3	Agricultural Production_crop_level_3
Agricultural Subsidy Card	—	Form is not nested
Bad Shocks	1	BadShocks_event_repeat_level_2
Basic Information	—	Form is not nested
Climate Event	—	Form is not nested
Crops	1	Crops_plot_repeat_begin_level_2
	2	Crops_crop_level_3
	3	Crops_group_crop_i_croptype_level_4
Current Migrants	1	Current Migrants_begin_repeat_mig_level_2
Drinking Water	1	Drinking Water_source_level_2
Employment	1	employment_work_done_level_2
Facilities	1	Facilities_facility_repeat_level_2
Farm Labor	1	farm Labor_begin_repeat_plot_level_2
	2	farm Labor_labor_repeat_level_3
Fertilizers And Pesticides	1	Fertilizers And Pesticides_plot_repeat_begin_level_2
	2	Fertilizers And Pesticides_crop_level_3
	3	FertilizersAndPesticides_group_crop_i_croptype_level_4
	4	Fertilizers And Pesticides_rep_fert_level_5
Fish Pond Inputs	1	Fish Pond Inputs_begin_level_2
Fish Pond Production	1	Fish Pond Production_pond_begin_repeat_level_2.1
	2	Fish Pond Production_river_repeat_level_2.2
	3	Fish Pond Production_fish_repeat_level_3
Food Diary	1	Food Diary_food_level_2
	2	35. Fooddiary_food_foodtype_level_3
Food Storage Capacity	—	Form is not nested
Good Shocks	1	Good Shocks_event_repeat_level_2
Household Composition	1	Household Composition_members_level_2
Housingand Sanitation	—	Form is not nested
Illness	1	illness_was_ill_level_2
Information Tools	1	Information Tools_tech_level_2
	2	Information Tools_tech_use_level_3
Mobile Internet	1	Mobile Internet_app_use_level_2
Irrigation	1	Irrigation_plot_repeat_begin_level_2
	2	Irrigation_crop_level_3
	3	Irrigation_group_crop_i_crop_repeat_type_level_4
Latrine Use	—	Form is not nested
Latrine Walk	1	latrine Walk_begin_repeat_lat_level_2
Livestock	1	Livestock_animal_level_2
Loans	1	Loans_each_loan_level_2
Marketing	1	Marketing_sellrell_prod_repeat_level_2
Non Agricultural Enterprise	1	Non Agricultural Enterprise_buss_level_2
Cosmetics	1	Cosmetics_item_repeat_level_2
Fuel Lighting	1	Fuel Lighting_item_repeat_level_2
Transport Travel	1	Transport Travel_item_repeat_level_2
Washing Cleaning	1	Washing Cleaning_item_repeat_level_2
Other Income	1	Other Income_income_level_2
Plots	1	Plots_plot_level_2
Remittance In	1	Remittance In_begin_repeat_mig_level_2
Remittance Out	1	Remittance Out_begin_repeat_mig_level_2
Renting Tools MachAn	1	renting Tools MachAn_plot_repeat_begin_level_2
	2	renting Tools MachAn_crop_level_3
	3	renting Tools MachAn_group_crop_i_crop_repeat_type_level_4
Safety Program	1	Safety Program_event_repeat_level_2
Savings	1	Savings_each_savings_level_2
School Attendance	1	school Attendance_missed_school_level_2
Subjective Wellbeing	—	Form is not nested
Transportation Tools	1	Transportation Tools_transport_level_2
	2	Transportation Tools_transport_use_level_3
Tubewell Walk	—	Form is not nested
Work Animals	1	Work Animals_animals_level_2
	2	Work Animals_animals_use_level_3
